# Versatile cyanobacteria control the timing and extent of sulfide production in a Proterozoic analog microbial mat

**DOI:** 10.1038/s41396-020-0734-z

**Published:** 2020-08-07

**Authors:** Judith M. Klatt, Gonzalo V. Gomez-Saez, Steffi Meyer, Petra Pop Ristova, Pelin Yilmaz, Michael S. Granitsiotis, Jennifer L. Macalady, Gaute Lavik, Lubos Polerecky, Solveig I. Bühring

**Affiliations:** 1 Microsensor Group, Max Planck Institute for Marine Microbiology, Bremen, Germany; 2 Hydrothermal Geomicrobiology, MARUM, University of Bremen, Bremen, Germany; 3 Microbial Physiology Group, Max Planck Institute for Marine Microbiology, Bremen, Germany; 4 Research Unit Environmental Genomics, Helmholtz Zentrum Munich, Munich, Germany; 5 Department of Environmental Engineering, University of Patras, Agrinio, Greece; 6 Pennsylvania State University, University Park, State College, PA USA; 7 Biogeochemistry Group, Max Planck Institute for Marine Microbiology, Bremen, Germany; 8 Department of Earth Sciences—Geochemistry, Faculty of Geosciences, Utrecht University, Utrecht, The Netherlands; 9 Present Address: Alfred Wegener Institute—Helmholtz Centre for Polar and Marine Sciences, Bremerhaven, Germany; 10 Present Address: Thünen Institute of Baltic Sea Fisheries, Thünen Institute, Rostock, Germany; 11 Present Address: DOE, Joint Genome Institute, Lawerence Berkeley National Lab, Berkeley, CA USA

**Keywords:** Microbial ecology, Biogeochemistry, Water microbiology

## Abstract

Cyanobacterial mats were hotspots of biogeochemical cycling during the Precambrian. However, mechanisms that controlled O_2_ release by these ecosystems are poorly understood. In an analog to Proterozoic coastal ecosystems, the Frasassi sulfidic springs mats, we studied the regulation of oxygenic and sulfide-driven anoxygenic photosynthesis (OP and AP) in versatile cyanobacteria, and interactions with sulfur reducing bacteria (SRB). Using microsensors and stable isotope probing we found that dissolved organic carbon (DOC) released by OP fuels sulfide production, likely by a specialized SRB population. Increased sulfide fluxes were only stimulated after the cyanobacteria switched from AP to OP. O_2_ production triggered migration of large sulfur-oxidizing bacteria from the surface to underneath the cyanobacterial layer. The resultant sulfide shield tempered AP and allowed OP to occur for a longer duration over a diel cycle. The lack of cyanobacterial DOC supply to SRB during AP therefore maximized O_2_ export. This mechanism is unique to benthic ecosystems because transitions between metabolisms occur on the same time scale as solute transport to functionally distinct layers, with the rearrangement of the system by migration of microorganisms exaggerating the effect. Overall, cyanobacterial versatility disrupts the synergistic relationship between sulfide production and AP, and thus enhances diel O_2_ production.

## Introduction

The evolution of oxygenic photosynthesis (OP) by cyanobacteria was one of the major transformative events in the history of life and is responsible for the bounty of life on Earth as we know it today. O_2_ is the most favorable electron acceptor used for respiration by myriads of organisms, and its accumulation in the atmosphere considerably changed the surface chemistry of the Earth. There are, however, many open questions concerning the history of O_2_ on Earth. Particularly the long lag in the rise of atmospheric O_2_ levels after the first appearance of free oxygen signals in the geological record still defy holistic mechanistic explanation [1]. One of the most mysterious episodes in Earth’s history after the Great Oxidation Event (~2.3–2.6 billion years ago) is often referred to as the “boring billion” during the mid-end Proterozoic, during which the O_2_ level remained below 0.01–10% of modern levels for about 1 billion years before rising markedly in the Neoproterozoic Oxidation Event.

Realizing that the Proterozoic global O_2_ stasis—the “dullest time in Earth’s history” [2]—cannot be explained exclusively based on geochemical mechanisms, biologically nuanced models suggest that global pelagic O_2_ production might have been tempered due to competition between OP and sulfide- or iron-driven anoxygenic photosynthesis (AP) [3–5]. The conceptual model introduced by Johnston et al. [3] specifically assumes euxinic water column conditions and suggests that organic carbon produced during sulfide-driven AP would be unavailable for aerobic respiration in the uppermost water column and thus would stimulate sulfate reduction in the deeper oceans. This feedback would then further increase rates of sulfide-driven AP in disfavor of OP. Competition between AP and OP for light and/or nutrients might therefore have set into motion a series of biogeochemical cascades that sustained sulfidic oceans and tempered O_2_ production. Johnston et al.’s proposal emphasizes that the physiology and ecology of early microorganisms need to be considered to understand Earth-scale effects of microbial activity.

Sulfur-driven AP can be performed by specialized obligate anoxygenic phototrophs, as well as by cyanobacteria [6]. While there is some speculation that early cyanobacteria used electron donors in addition to sulfide and water [7], only reduced sulfur compounds have been confirmed as alternative electron donors in extant cyanobacteria [6, 8–11]. Sulfide:quinone reductase (SQR) is the only enzyme involved in cyanobacterial AP that has been identified so far, and might be the only equipment needed for light-driven sulfide oxidation [12]. Sulfide oxidation is integrated into the oxygenic photosynthetic electron transport chain such that AP and OP can be performed simultaneously and yet compete for their portion in the overall photosynthetic rate [13] (Fig. S1). Photosynthesis rates in individual organisms and the environment therefore reflect a competition between OP and AP. The outcome of this competition manifests itself in the partitioning between AP and OP, and is shaped by electron donor and light availability, and specific affinities of, e.g., enzymes of different microbes specialized in either one of these photosynthetic modes, or within versatile cyanobacteria capable of switching between them.

Although Johnston et al.’s model was developed to explain water column conditions, they also suggested that the same feedback mechanisms would arise in cyanobacterial mats. As opposed to pelagic systems, cyanobacterial mats are densely packed ecosystems shaped by diffusional transport and intense metabolic interactions occurring over only a few mm depth. The lack of stratification disturbance by advective exchange of sulfur compounds, organic carbon, and nutrients, might have favored the establishment of a positive feedback loop. On modern Earth, mats often form under extreme conditions, such as high salinities, that provide natural protection from the majority of grazing animals. Proterozoic cyanobacterial mats, however, flourished in a vast proportion of the sunlit shallow seafloor, under low-O_2_ and high reductant conditions even after the first global rise of atmospheric O_2_ [3]. Among these reductants, reduced sulfur compounds might have played a central role as electron donors for AP. Phylogenetic as well as sulfur isotope data [14] suggest that even the earliest microbial mats might have been characterized by intense sulfur cycling despite limited external sulfur input [15–17]. Predominantly ferruginous ocean conditions are nowadays a widely accepted model [17–20] replacing the former model of a global euxinic ocean [21]. This questions the applicability of the Johnston et al.’s model beyond highly productive coastal environments that might have represented the local exception and were likely euxinic [1]. Thus, the model by Johnston et al. might rather apply to the microbial ecosystems that flourished and evolved under sunlit sulfidic conditions: cyanobacterial mats.

This study aimed to test if the open ocean model of positive feedbacks between sulfide-driven AP and sulfate reduction [3] can be validated in an analog ecosystem to coastal mats of the Proterozoic characterized by cyanobacterial AP. Such analog mat ecosystem flourishes under diverse sulfide to O_2 _ratios along the flow path of sulfidic spring water emerging from the Frasassi cave system, Italy [22, 23]. Using an approach that combines microsensor measurements and stable isotope probing (SIP), we assessed rates of AP, OP and sulfide production, and followed the fate of assimilated CO_2_ over simulated diel cycles.

## Methods

### Experimental design

To enable simultaneous assessment of depth-resolved gross rates of light-driven sulfide consumption and O_2_ production, as well as the fate of freshly produced dissolved organic carbon (DOC), we sampled a cyanobacterial mat without the underlying sediment from the Frasassi sulfidic springs in September 2012 (Fig. S2). The mat was placed in a flow chamber that accommodated sufficient area for microsensor measurements and sub-sampling of the mat during defined conditions (Fig. S3) that are detailed in the following sections. The incubation started with exposure to darkness for 8 h. ^13^C-bicarbonate solution was added to the water column and to a spring water reservoir underneath the mat after ~5.5 h. During the following stepwise increase of light intensity (7, 19, 89, and 315 µmol photons m^−2^ s^−1^), net and gross rates of AP and OP were continuously monitored using microsensors in three replicate spots of the mat. Light intensity was only increased after a steady state had established for at least 30 min (determined from concentration depth profiles). Triplicate subsamples (1 cm^2^) of the mat were taken in regular intervals over the course of the experiment to (1) determine bulk rates of inorganic carbon assimilation, (2) identify the functional groups involved in this ^13^C assimilation based on fatty acids (FA), (3) follow the flow of assimilated carbon into the ^13^C-DOC pool, and (4) monitor changes in the active community based on 16S rRNA sequencing. To be able to differentiate between the effect of light intensity and photosynthetic O_2_ production, after exposure to 315 µmol photons m^−2^ s^−1^, DCMU (3-(3,4-dichlorophenyl)-1,1-dimethylurea; dissolved in ethanol), an inhibitor of OP [24], was added to the water column in the dark to a final concentration of ~10 µM. The mat was then again exposed to 315 µmol photons m^−2^ s^−1^ for 8 h. In a second incubation run with fresh mat material DCMU was added in the beginning, before addition of ^13^C-bicarbonate.

### Sampling and setup

The cyanobacterial mat forms along the flow path of “Main Spring” that emerges from the Frasassi cave system (Fig. S2, 43°24′4″N, 12°57′56″E, [23]). The day before first mat sampling, water column samples for total sulfide determination were collected and conserved in 2% zinc acetate solution. Concentration was assessed on the same day according to Cline [25]. O_2_ concentration and pH were determined using microsensors (see below). Temperature at the mat surface was measured with a PT1000 mini-sensor (Umweltsensortechnik, Geschwenda, Germany). Spring water was collected from the outflow of main spring and transported to the laboratory facilities of the Osservatorio Geologico di Coldigioco (~45 min driving time) and immediately prepared for use in the flow chamber.

The flow chamber was a larger version of what is described in [26] (Fig. S3). Briefly, the upper part of the flow chamber was separated from a bottom chamber using fibrous web and GF/F filters. The bottom chamber was filled with HEPES-buffered (pH 7.2) spring water that was then purged with N_2_ using needles penetrating the rubber stoppers on the wall of the chamber. The upper flow chamber was connected with tubing via five inlets to a water pump in a thermostated 20 L recycle of freshly sampled N_2_-bubbled spring water.

The following day, a 30 × 40 cm piece of mat was carefully lifted off the sediment, transferred into a plastic container, and transported cooled and in the dark to the laboratory. A small subsection of the mat was flash-frozen for 16S rRNA analysis on site. Upon arrival in Coldigioco, the mat was immediately placed onto the GF/F filters in the flow chamber. Neutralized Na_2_S was slowly added to the 20 L recycle of the flow cell. After ~6 h of dark incubation, ^12^C- and ^13^C-sodium bicarbonate (^13^C-DIC final atom fraction of ≈6%) were injected into the bottom chamber and briefly stirred. Subsequently, ^12^C- and ^13^C-sodium bicarbonate (^13^C-DIC final atom fraction of ≈6%) was added to the recycle. To allow for homogeneous distribution of the label, the pumping speed was increased for 5–10 min. To minimize outgassing of H_2_S and exchange of ^13^CO_2_ with the atmosphere, the spring water in the 20 L recycle was covered with paraffin oil and the water column in the flow cell was covered with transparent plastic wrap. Small holes were kept in the wrap to allow microsensor measurements. Immediately after bicarbonate addition, the first mat and water column samples were taken. Homogenous illumination was achieved by using two large cold-white lamps (Envirolite), the distance of which to the mat was adjusted to change light conditions. Incident irradiance at the mat surface was determined using a cosine‐corrected quantum sensor connected to a LI‐250A light meter (both LI‐COR Biosciences GmbH, Germany).

### Microsensors

O_2_, H_2_S, and pH microsensors with a tip diameter of 10, 20, and 50 µm, respectively, and response time of <2 s were constructed and calibrated as described previously [22, 27–29]. All sensors were mounted on a multi-sensor holder and the tips were separated by less than 1 cm. The motorized positioner for vertical microprofiling was mounted on a horizontal motorized positioner, which allowed automated and reproducible repositioning of the sensors in three replicate spots during the incubation. At each light condition H_2_S, pH, and O_2_ depth profiles were measured in the three spots. After correction for the measurement angle, depth resolution of profiling was ~450 µm in the water column and depths greater than 4 mm, and ~180 µm in the uppermost 4 mm of the mat. Total sulfide (S_tot_) concentration (∑[S^2−^, HS^−^, H_2_S]) was calculated from H_2_S concentration and pH. When steady state was reached at each light intensity, gross photosynthesis rates over depth in one of the replicate spots was measured using the previously described O_2_- and H_2_S-based light-dark shift methods for OP and AP, respectively [22]. Fluxes and local volumetric net rates of production/consumption were calculated from concentration depth profiles using Fick’s first and second law of diffusion, respectively, using diffusion coefficients corrected for temperature and salinity (1.35 × 10^−5^ cm^2^ s^−1^ for sulfide and 1.78 × 10^−5^ cm^2^ s^−1^ for O_2_).

To be able to compare rates of OP, AP, and chemosynthetic sulfide oxidation to rates derived from the SIP assays, we calculated potential C-fixation rates. For OP we multiplied the depth-integrated gross rates of O_2_ production with a factor 1 assuming the stoichiometry:

1 $${\mathrm{H}}_{\mathrm{2}}{\mathrm{O}} + {\mathrm{CO}}_2 \to {\mathrm{O}}_2 + {\mathrm{CH}}_{\mathrm{2}}{\mathrm{O}}.$$

For AP we took a similar approach and multiplied the depth-integrated gross rates of light-dependent sulfide consumption by a factor 2 assuming sulfide oxidation to zero-valent sulfur according to:

2 $${\mathrm{2H}}_{\mathrm{2}}{\mathrm{S}} + {\mathrm{CO}}_2 \to 2{\mathrm{S}}^{\mathrm{0}} + {\mathrm{CH}}_{\mathrm{2}}{\mathrm{O}} + {\mathrm{H}}_{\mathrm{2}}{\mathrm{O}},$$

as previously described in [13, 22]. The rate of predicted CO_2_ fixation by sulfur-oxidizing bacteria (SOB) was estimated based on the fluxes of sulfide and O_2_ into the zone of aerobic sulfide oxidation, and the previously determined energy conservation efficiency of 16.9% for autotrophic aerobic sulfide oxidation in Frasassi mats [22, 30]. The end member stoichiometries for predominant oxidation of sulfide to S^0^ and SO_4_^2−^ under the incubation conditions follow, respectively:

3 $${\mathrm{H}}_{\mathrm{2}}{\mathrm{S}} + 0.4{\mathrm{O}}_{\mathrm{2}} + 0.1{\mathrm{CO}}_2 \to 1{\mathrm{S}}^0 + 0.1{\mathrm{CH}}_2{\mathrm{O}} + 0.9{\mathrm{H}}_{\mathrm{2}}{\mathrm{O}},$$

and

4 $${\mathrm{H}}_{\mathrm{2}}{\mathrm{S}} + 1.5{\mathrm{O}}_{\mathrm{2}} + 0.5{\mathrm{CO}}_2 + 0.5{\mathrm{H}}_{\mathrm{2}}{\mathrm{O}} \to 1{\mathrm{SO}}_4^{2 - } + 0.5{\mathrm{CH}}_2{\mathrm{O}} + 2{\mathrm{H}}^ +.$$

The stoichiometry was adjusted according to the concentrations of H_2_S and O_2_, and pH for each time point assuming a constant thermodynamic efficiency but variable products of sulfide oxidation (Table S1).

### CO_2_ assimilation rates

To determine the ^13^C/^12^C of the dissolved inorganic carbon (DIC) pool, water column, and bottom chamber water samples were taken in regular intervals during the incubation and preserved by addition of HgCl_2_ and ZnCl_2_ in Exetainers (Labco, UK) without headspace. The ^13^C/^12^C ratio was determined by isotope-ratio-monitoring gas chromatography–mass spectrometry (GC-MS) (VG Optima; Micromass, Manchester, UK) [31].

The ^13^C/^12^C in the mat sampled during the incubation was determined using an automated elemental analyzer (FlashEA, 1112 series) coupled to a Delta Plus Advantage mass spectrometer (Finnigan DeltaplusXP, both from Thermo Scientific) after freeze-drying and decalcification with ortho-phosphoric acid. The leftovers of freeze-dried samples were pooled and used for FA-SIP and DOC extraction. Total CO_2_ fluxes were calculated as the rate of increase in the isotopic labeling of the mat, considering the average areal weight of mat and correcting for the labeling of the DIC pool.

### ^13^C-DOC

To estimate the ^13^C/^12^C of the DOC pool, the remaining freeze-dried and decalcified mat material was pooled for each time point and 1.5 mL of ultrapure water were added to each sample. The re-suspended mat material was vigorously shaken. After centrifugation, the supernatant was filtered through 0.45 µm PES syringe filters into 2 mL septum vials (Zinsser). To convert the DOC into CO_2_, we followed the approach of Menzel and Vaccaro [32, 33] by adding 30 mg potassium persulfate and 60 µL 3% ortho-phosphoric acid before autoclaving for 1.5 h. The ^13^C/^12^C ratio in the resultant CO_2_ pool in the headspace was determined with isotope-ratio-monitoring GC-MS (VG Optima; Micromass, Manchester, UK). ^13^C-Glucose was used as a standard to assess conversion efficiency. As the efficiency of conversion into CO_2_, however, likely varies amongst different compounds of the DOC pool, we did not aim to quantify DOC but only report the relative changes of the ^13^C/^12^C-DOC.

### FA-SIP

The total lipids extracts (TLE) of freeze-dried mat samples were obtained following the procedure in [34], with modifications (see Supplementary material). Elemental sulfur was removed from the TLE using copper powder (Sigma-Aldrich), activated with 4 N HCl as explained in [35]. An aliquot of the TLE was saponified according to [36]. Prior to analysis, FAs were derivatized using boron trifluoride (BF_3_) in methanol (Merck), leading to FA methylesters.

FAs were identified by coupled GC-MS (Agilent 6890N GC with Agilent 5973N mass selective detector). Quantification was done by GC coupled to a flame ionization detector using squalene as injection standard. The carbon isotopic compositions were determined by GC-isotopic ratio-MS using a Thermo Scientific Trace GC Ultra coupled to a Thermo Scientific Delta V Plus IRMS. The carbon isotope ratios were expressed in the delta notation (δ^13^C), based on which the relative increase of label (Δδ^13^C) was calculated by subtracting the δ^13^C of each FA at the first time point [37].

FA were classified into cyanobacterial-FA, sulfur reducing bacterial (SRB)-FA and SOB-FA according to the literature. Cyanobacterial-FA included the even-numbered monounsaturated C_16:1ω9_ and C_18:1ω9_, and the polyunsaturated FA C_16:2_ and C_18:2_ [37–41]. SRB-FA included *ai*C_15:0_, *i*C_15:0_, *10Me-*C_16:0_, *ai*C_17:0_, *i*C_17:0_, and *i*C_17:1_, as well as C_15:1_, C_17:0_, and C_17:1_ [42–45]. SOB-FA included the even-numbered monounsaturated C_16:1ω7_ and C_18:1ω7_ [46–48].

Relative ^13^C uptake rate contribution of FAs (FA-RUR) for each interval between time points were calculated as the ratio of the rate of increase in the isotopic labeling of each SRB-FA (δ^13^C) over time (*t*) and the total rate of increase of all SRB-FAs over time, as:

5 $$\mathrm{FA -RUR} = \frac{{\frac{{\Delta \delta ^{13}\mathrm{C}}}{{\Delta t}}}}{{\Sigma \frac{{\Delta \delta ^{13}\mathrm{C}}}{{\Delta t}}}}.$$

We then clustered sub-groups of SRB-FAs according to the patterns of increase/decrease in rate (examples in Fig. S4a, b), with group 1 (SRB-FA1) comprising *10Me-*C_16:0_ and *i*C_17:1_, group 2 (SRB-FA2) comprising *ai*C_17:0_, *i*C_17:0_, and C_17:1_ and group 3 (SRB-FA3) comprising *ai*C_15:0_, *i*C_15:0_, C_15:1_, and C_17:0_.

### 16S rRNA extraction and sequencing

The mat sample taken directly in main spring, and six of the subsamples taken during the flow chamber incubations were chosen for sequencing. RNA was extracted from these seven RNAlater stabilized subsamples with FAST RNA Pro Soil direct kit (MP Bio). Pyrosequencing libraries were constructed as described previously [49] with modifications (see Supplementary information). Emulsion PCR, emulsion braking and sequencing were performed applying the GS FLX Titanium chemistry following the supplier’s protocols (Roche).

### 16S rRNA analysis

The raw sequencing data sets were initially processed with the next-generation sequencing analysis pipeline of the SILVA project (available at www.arb-silva.de/ngs) [50] to obtain sequence and alignment quality-based filtering of the amplicons, aligned sequences, and a taxonomic classification (see Supplementary information). Based on quality filtering, a subset of cyanobacterial and deltaproteobacterial sequencing reads were selected for further oligotyping analysis using Oligotyping version 2.1 (available from https://github.com/merenlab/oligotyping). Oligotype representatives were then added to the SILVA RefNR 132 guide phylogenetic tree using the ARB-parsimony addition tool [51] (Table S2). Further processing of oligotype data was performed in R environment for statistical computing (https://www.R-project.org/), using package phyloseq (version: 1.19.1) [52] (see Supplementary information). Sequence data has been deposited in the European Nucleotide Archive (ENA) at EMBL-EBI under accession number PRJEB38493 [53] (see Supplementary information).

## Results

### Mat is dominated by filamentous cyanobacteria and SOB

**Fig. 1:**
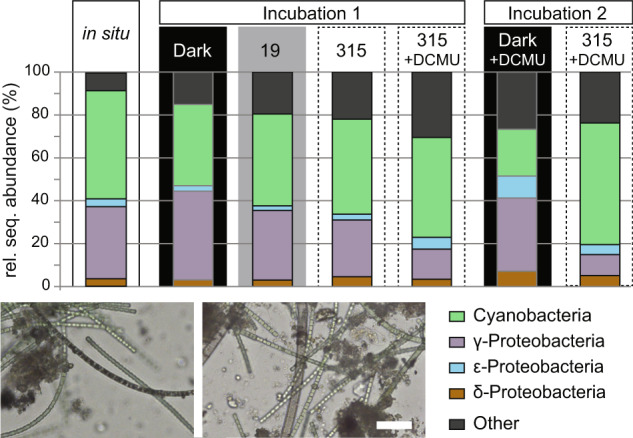
Relative abundance of 16S rRNA sequences at the phylum level and light microscopic images of cyanobacterial and SOB filaments in mat subsamples taken in main spring and during the incubations. Incubation 1 was initially run without DCMU addition, while it was added to the flow chamber in the beginning of incubation 2. Subsamples for microscopic images shown here were taken in the beginning of incubation 1, before addition of labeled bicarbonate. Scale bar in the right image is 20 µm.

Cyanobacterial and gammaproteobacterial 16S rRNA sequences dominated throughout all samples taken directly in the spring and during the incubations, which was consistent with highly abundant filamentous cyanobacteria and large sulfur bacteria observed by microscopy (Fig. 1). Interestingly, relative 16S rRNA sequence abundance was <0.5% for potential obligate anoxygenic phototrophs (e.g., *Chloroflexaceae*, *Chlorobi* and purple bacteria) and suspicious morphotypes, such as *Chromatiaceae*, were not observed visually (data not shown).

### Transitions between anoxygenic and oxygenic photosynthesis depend on irradiance

**Fig. 2:**
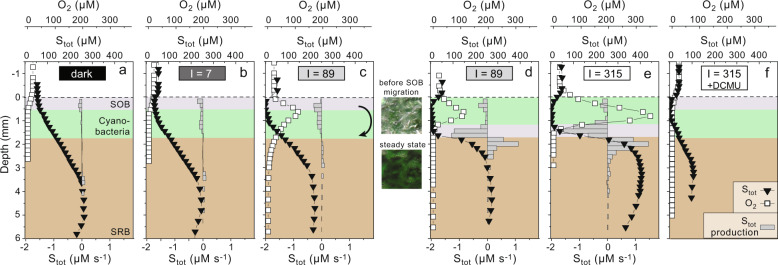
Depth profiles of O_2_ and S_tot_, and volumetric rates of S_tot_ production and consumption, measured during the first incubation of cyanobacterial mat in the flow chamber exposed to variable light conditions (*I* = intensity in µmol photons m^−2^ s^−1^). All profiles  in (**a**–**b**) and (**d**–**e**) were measured at the end of the exposure time to a certain light condition and correspond to a steady state, except for the profiles in (**c**), which were obtained just before migration of SOB, and profiles in (**f**), which were measured during the continuous S_tot_ decrease (Fig. 3b). Photos of the mat taken before and after migration illustrate the change of mat appearance from whitish to dark green. The vertical mat structure is approximated behind the profiles.

In the dark and after adjustment of pH and O_2_ and total sulfide (S_tot_) concentration in the water column approximately to in situ conditions (Table S3), the mat surface was covered with SOB as deduced from the whitish appearance of the mat (see inset between Fig. 2c, d). Consistent with this observation, microsensor profiling revealed concave-shaped steady-state depth profiles of O_2_ and S_tot_ concentrations in the top 500 µm of the mat, indicative of consumption, i.e., aerobic sulfide oxidation, in this zone (Fig. 2a). Upward sulfide fluxes from deep layers into the sulfide oxidation zone in the three replicate spots were in the same range as measured in situ [22] even though the sample consisted of the thin mats only (~5–6 mm), without underlying sediment or any artificially added sulfide supply from underneath. This indicated within-mat production of sulfide between ~3–5 mm depth, which is supported by the convex shape of the steady-state S_tot_ profiles and corresponding positive local volumetric rates in this zone (Fig. 2a).

**Fig. 3:**
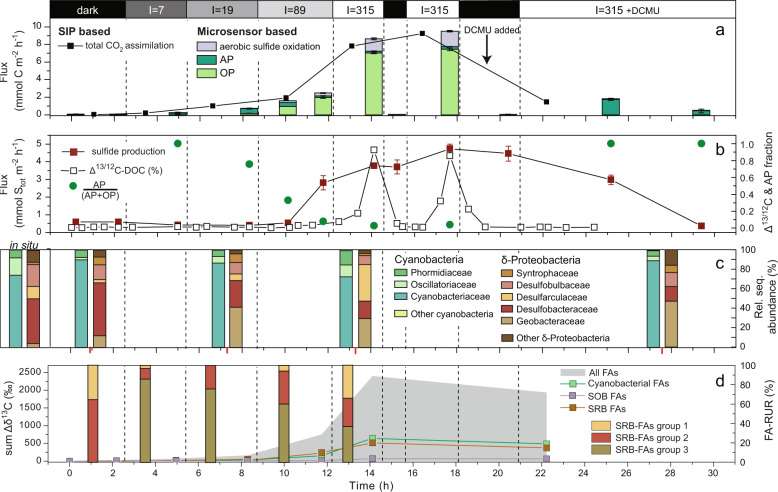
Results of the first incubation of cyanobacterial mat in the flow chamber exposed to variable light conditions indicated on the top panel (*I* = intensity in µmol photons m^−2^ s^−1^). **a** Depth-integrated gross rates of AP and OP calculated based on the light-induced dynamics of S_tot_ and O_2_, respectively, measured in the cyanobacterial layer during steady state in the same mat spot where also profiles in Fig. 2 were measured. Values were converted to predicted CO_2_ fixation rates and are represented as stacked bars. Also, fluxes of CO_2_ derived from ^13^C-bicarbonate assimilation (see raw data in Fig. S5) are shown. Injection of DCMU is indicated by black vertical arrow. **b** ^13^C/^12^C of the porewater DOC pool of the mat samples relative to the isotopic ratio at the first time point, areal sulfide production rate, and the relative contribution of AP to total photosynthesis (AP + OP) calculated from data in (**a**). **c** Relative abundance of 16S rRNA sequences in the four mat subsamples taken during the incubation. The bar charts represent closer views into the phyla in Fig. 1. Transcription patterns amongst the deltaproteobacteria (right bars) and cyanobacteria (left bars) are shown, with the red mark on the *x*-axis marking the time of sample collection. **d** Incorporation of ^13^C into all fatty acids, and the fraction assigned to cyanobacteria, SOB, and SRB relative to the sample at the first time point, and relative uptake rates into the three sub-groups of SRB-FAs (FA-RUR).

Upon exposure to an incident irradiance of 7 µmol photons m^−2^ s^−1^, the zone of S_tot_ consumption expanded to the cyanobacterial layer at depths 0.3–1.1 mm (Fig. 2b). Depth-integrated gross rates of AP (light-driven sulfide consumption; Fig. 3a) matched the net sulfide flux consumed in the cyanobacterial layer, the latter calculated from the steady-state S_tot_ depth profile as the difference between the fluxes of S_tot_ at the upper and lower boundary of the cyanobacterial layer (Table S1). This suggests that sulfide consumption in the cyanobacterial layer was predominantly driven by AP, and that there was negligible local source of sulfide. Sulfide was not entirely depleted, which implies that AP was limited by light, and not by electron donor (Fig. 2b).

Upon exposure to 89 µmol photons m^−2^ s^−1^, light-driven sulfide consumption rates increased sufficiently to entirely deplete sulfide underneath the SOB layer (Fig. 2c). As a result, the cyanobacteria additionally performed OP, consistent with the physiology of versatile cyanobacteria (Fig. S1) [13, 54]. After this transition between photosynthetic modes, downward migration of the SOB from the mat surface to underneath the cyanobacterial layer occurred (Fig. 2d). Since the presence of sulfur granules inside SOB leads to intense light scattering (which gives SOB populations their characteristic white appearance), this downward migration of the SOB population resulted in a locally increased availability of light in the cyanobacterial layer [22], which led to an increase in the rate of gross OP (compare OP rates at time 10.1–11.7 h in Fig. 3a, and the corresponding bar profiles in Fig. 2c, d). After disappearance of the white layer from the mat surface, we observed a distinct zone of aerobic sulfide oxidation underneath the photosynthetically active layer. In this zone, we did not detect light-induced dynamics of sulfide. The upward sulfide flux was consumed with the photosynthetically produced oxygen at a S_tot_:O_2_ consumption ratio of ~2.3 suggesting predominantly incomplete sulfide oxidation to zero-valent sulfur (characterized by a relatively low carbon yield of ~0.12 mol C (mol S)^−1^) [30] (Table S1). As the sulfide flux from underneath did not reach the cyanobacterial layer, AP was almost exclusively fueled by sulfide from the water column (Table S1). Furthermore, because the available sulfide in the overlying water was lower than that from below, the depth-integrated rate of AP decreased substantially.

Exposure to irradiance of 315 µmol photons m^−2^ s^−1^ led to a further increase in the rate of OP but no significant change in AP (Figs. 2e and 3a). Driven by photosynthetically produced O_2_ from the cyanobacterial layer, the rate of O_2_ consumption increased. Therefore also the predicted CO_2_ fixation rate, by aerobic sulfide oxidation in the SOB layer, increased (Fig. 3a). The ratio of sulfide and O_2_ consumption by this process decreased from ~2.3 to ~0.85 suggesting a switch from incomplete to predominantly complete aerobic sulfide oxidation to sulfate [30] (Table S1).

**Fig. 4:**
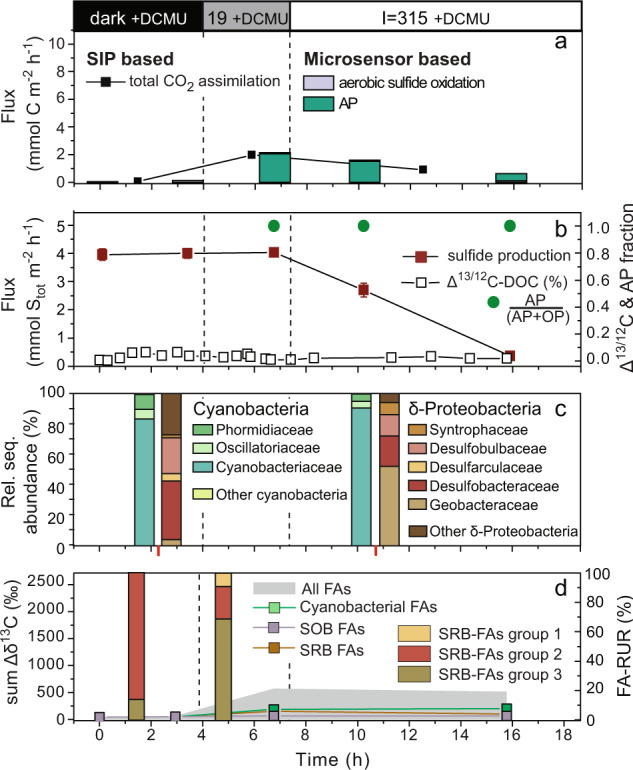
Results of second incubation of cyanobacterial mat in the flow chamber. **a**–**d** are the same as in Fig. 3. DCMU was added ~1 h before the start of measurements and sub-sampling.

After addition of DCMU, OP was inhibited and only AP was detectable (Figs. 2f and 3a). O_2_ only penetrated down to a maximum of ~0.5 mm below the mat surface. As a result, the SOB returned to the surface despite continued illumination with 315 µmol photons m^−2^ s^−1^. This behavior was unexpected because of reports of photophobic responses of *Beggiatoa* [55]. Similarly, in a separate run of the experiment with DCMU addition at the beginning of the diel cycle (Fig. 4), the SOB did not migrate despite exposure to light intensities >300 µmol photons m^−2^ s^−1^. This is consistent with the shallow penetration of O_2_ (Fig. 2f) due to the lack of O_2_ production in the mat by cyanobacterial OP.

^13^C-bicarbonate was assimilated into mat biomass throughout the initial dark phase and in the light (Fig. S5), at rates increasing with light intensity (Fig. 3a). ^13^C/^12^C values of the replicate samples were very similar (error bars in Fig. S5) suggesting horizontally homogeneous mat activity. This is supported by the high reproducibility of fluxes calculated from the measurements in the three replicate spots (Table S1). Total CO_2_ assimilation rates in the absence and presence of DCMU (Figs. 3a and 4a) deviated by up to 70% from the C-fixation rates estimated by summing the depth-integrated rates of gross AP, OP, and aerobic sulfide oxidation under dark and low light conditions. These large differences are likely caused by relative uncertainties increasing with decreasing rates. C-fixation rates for irradiances ≥89 µmol photons m^−2^ s^−1^ were 90–97% of the microsensor-based rates, supporting our assumption that in versatile cyanobacteria sulfide oxidation contributes to the photosynthetic electron chain with two electrons and follows Eq. (2) for CO_2_ fixation.

**Fig. 5:**
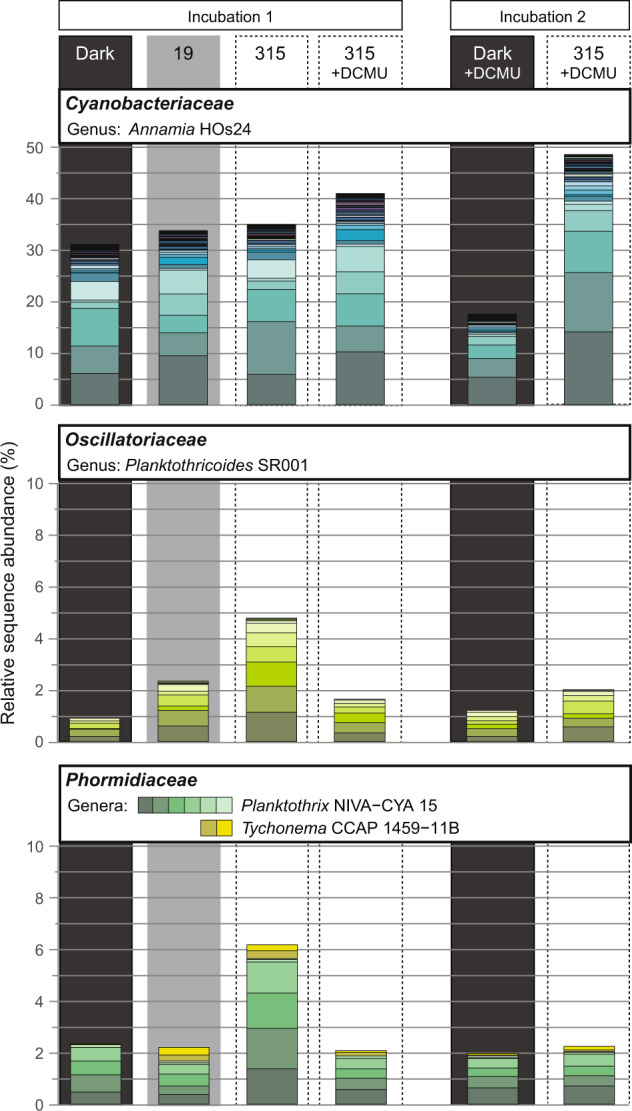
Oligotyping results of first and second incubation run. Incubation conditions (19 and 315 µmol photons m^−2^ s^−1^; with and without DCMU) are indicated in the top panel. Note that the *Annamia* oligotypes amongst the *Cyanobacteraceae* dominate throughout the experiment. Other oligotypes only contributed substantially to the pool of transcribed 16S rRNA at 315 µmol photons m^−2^ s^−1^, when oxygenic photosynthesis dominated (see Fig. 3).

Despite the long incubation time, shifts between photosynthetic modes (see AP:(OP + AP) ratio in Fig. 3b), and the use of the inhibitor DCMU, the cyanobacterial rRNA sequences were dominated by *Cyanobacteriaceae* (Figs. 3c and 4c) throughout the experiment. Most sequences were affiliated with the genus *Annamia* (Fig. 5). Upon transition from dark to AP-dominated conditions, the relative abundance of oligotypes affiliated with *Annamia* and *Planktothricoides* SR001 (*Oscillatoriaceae*) increased, particularly in incubation 2, while the abundance of other cyanobacterial oligotypes remained low (Fig. 5). However, during OP-dominated conditions, the balance between families and genera in the transcribed cyanobacterial rRNA pool changed (Fig. 3c and 5). Namely, we observed relatively higher rRNA transcription by *Phormidiaceae* (genera *Planktothrix* NIVA-CYA 15 and *Tychonema* CCAP 1459–11B) and *Planktothricoides* SR001, with the *Planktothrix* rRNA sequences affiliating with the isolate *Planktothrix* FS34 derived from the sample site in the Frasassi Gorge [26] (Figs. 3c, 5, and S6).

The relative abundance of transcribed rRNA sequences of potential chemotrophic SOB among the gamma- and epsilonproteobacteria decreased slightly with incubation time (Fig. S7). Yet, sequences affiliating with *Beggiatoaceae* dominated throughout the experiment. After extended exposure to DCMU, however, we consistently observed increased epsilonproteobacterial rRNA transcription, relative to gammaproteobacterial (Fig. S7).

### Sulfide production varies with photosynthetic mode

Within-mat sulfide production in the zone underneath the autotrophic layers was estimated as the difference between the steady-state upward flux of sulfide into the SOB/cyanobacteria layer and of the downward flux into the bottom chamber (Fig. 3b) and is additionally illustrated by the local volumetric rates in Fig. 2 (bars). Intriguingly, sulfide production responded to changes in light intensity. In the dark, sulfide fluxes were constant for ~8 h (Fig. 3b, Table S1) and also the sulfide concentration in the zone of production (below ~3 mm; Fig. 2a) did not change significantly. Upon exposure to low light, when photosynthesis was predominantly anoxygenic, the rate of sulfide production started to decrease (Figs. 3b and 2b, c). When the cyanobacteria switched to OP during exposure to higher light, areal sulfide production rates increased again (Fig. 3b). Also, the change in curvature of the profiles and corresponding volumetric rates suggested that the zone of sulfide production moved to a shallower depth or that an additional source of sulfide closer to the cyanobacteria became active (Fig. 2d, e). When OP was inhibited by DCMU, sulfide concentration decreased rapidly (Figs. 2f, 3b, and 4b). Calculation of volumetric rates was omitted because concentration profiles did not approach steady state due to the continuous decrease of sulfide concentration. Consequently, rates of AP also decreased due to the limited supply of the electron donor. Ultimately, AP and aerobic sulfide oxidation driven by an internal sulfur cycle could not be maintained in the presence of DCMU and mat autotrophy was entirely dependent on the external supply of electron donor from the overlying water column. Before the disrupting event of DCMU addition, process rates were reproducible throughout the experiment, which indicates maintained activity and biomass of the microbial key players in the sulfur and carbon cycle (compare rates in the three dark phases, and two phases of exposure to 315 µmol photons m^−2^ s^−1^ in Fig. 3 and Table S1). The decrease in AP and sulfide production upon inhibition of OP, however, is expected to finally lead to mat community collapse and biomass degradation, as already indicated by reduced relative abundance of transcribed 16S rRNA of the potential autotrophic key players (Fig. 1).

### A link between organic carbon release by cyanobacteria and sulfide production

The ^13^C/^12^C of the DOC pool increased with increasing OP (Fig. 3b) suggesting accumulation of freshly assimilated, likely excreted, soluble organic carbon in the layer of production due to insufficient local sink strength. This ^13^C-enriched DOC rapidly disappeared in the dark, which implies net oxidation or assimilation into nonsoluble biomass components. Whenever AP-dominated total photosynthesis (low light conditions and DCMU treatment), no net increase in the ^13^C/^12^C of the DOC pool was detected (Figs. 3b and 4b). We would therefore expect sulfide production (see above) to be tightly linked to the ^13^C-DOC release. Consistent with this idea, the ^13^C/^12^C of the FA pool characteristic of cyanobacteria and SRB increased concomitantly (Fig. 3d). Taken together, dynamics of S_tot_ production, ^13^C-DOC and ^13^C-FAs support the concept that the highly active OP-performing cyanobacteria assimilate ^13^C into biomass including FAs, but also excrete labile freshly assimilated DOC that is rapidly transferred to SRB to be incorporated into their FAs. The relative uptake into the two SRB-FA groups (FA-RUR, Figs. 3d and 4d, bar chart) showed that during AP the relative contribution to the rate of group 3 was highest, while group 1 only showed significant contribution during the OP phase.

**Fig. 6:**
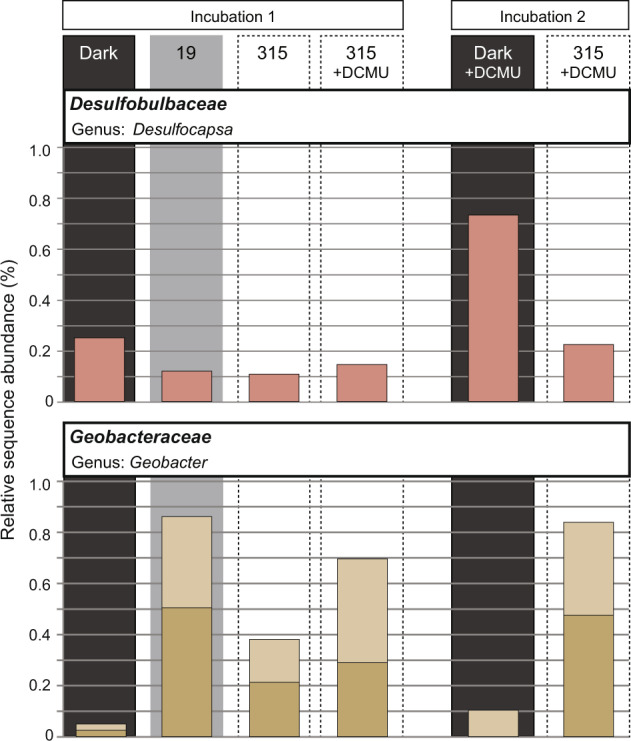
Oligotyping results of first and second incubation run for deltaproteobacterial sequences. Incubation conditions (dark; 19 and 315 µmol photons m^−2^ s^−1^; with and without DCMU) are indicated in the top panel.

Changes in sulfide production rates were accompanied by changes in the transcribed deltaproteobacterial rRNA sequences (right bars in Figs. 3c and 4c). In the dark, *Desulfobacteraceae* dominated while under AP conditions diversity increased, with *Geobacteraceae* taking over (Figs. 3c, 4c, and 6). *Desarfarculaceae* rRNA was only abundant under OP conditions. The change in SRB rRNA abundances closely tracked changes observed among the relative uptake rates into SRB-FAs (compare Figs. 3c, d and 4c, d). Overall, this indicates that the deltaproteobacterial community responds to changes in cyanobacterial metabolism. The differential stimulation of activity is likely driven by changes in source and composition of DOC, and/or due to the variable supply of oxidized sulfur compounds by AP and aerobic sulfide oxidation, i.e., changes in the abundance of intermediate sulfur species and sulfate.

## Discussion

We can paint a picture of a cyanobacterial mat that has spatially strongly separated zones of aerobic sulfide oxidation, photosynthesis, and sulfide production. Yet, we observed well-tuned coupling of these processes in our experiments. The network of metabolic coupling changed dramatically with light conditions, i.e., the diel light cycle, and the cascade of changes was controlled by metabolic transitions of individual functional groups, migration behavior, and rate of solute transport.

In the dark, the cyanobacterial community was sandwiched between sulfide consumers (SOB) and sulfide producers (SRB). Low light and high local sulfide concentration favor AP over OP in versatile cyanobacteria (Fig. S1), consistent with OP rates below detection limit. Predominant AP in the cyanobacterial layer did not induce a change of mat structure. However, as photosynthesis transitioned from AP to OP at higher light, a cascade of feedback effects was initiated. Most prominently, the transition to OP was followed by a downward migration of SOB. The relative abundance of transcribed rRNA levels of potential SOB amongst the Gammaproteobacteria only changed substantially after prolonged exposure of the mat to DCMU (Fig. S7). Thus, the short-term changes in the depth of sulfide oxidation and the S_tot_:O_2_ consumption ratio were likely only driven by migration and adjustment of the sulfide oxidation product (zero-valent sulfur vs. sulfate) depending on the availability of S_tot_ and O_2_ [22, 30], and not by a shift in active community. As migration was not observed upon exposure to similar light intensities in the presence of DCMU, its direct cause was the photosynthetically produced O_2_ rather than the light. Specifically, the SOB are characterized by a fixed efficiency of energy conversion and are thus restricted to the consumption of O_2_ and S_tot_ in a specific range of ratios [30]. They likely migrated below the cyanobacterial layer because remaining on top would not have allowed them to adjust their O_2_:S_tot_ consumption ratio to match the stoichiometry of the sulfide and O_2_ fluxes imposed by their environment [30]. A deviation from the possible range of stoichiometries implies that O_2_ cannot be consumed within the SOB layer. The resultant local increase in O_2_ concentration likely stimulates a phobic response and the migration [56]. The disappearance of the white, light-scattering SOB cover shading the cyanobacteria led to an increased availability of light for, and thus to increased rates of cyanobacterial OP. This cascade of transitions is beneficial for both cyanobacteria and SOB: the SOB have direct access to sulfide from underneath and photosynthetically produced O_2_, while the cyanobacteria can fully exploit the photon flux at the surface.

Upon transition to OP, diversity of transcribed cyanobacterial rRNA increased. Amongst the increasing sequences, we identified *Planktothrix* oligotypes affiliating with *Planktothrix* FS34 (Fig. S6). This cyanobacterium was isolated from the Frasassi sulfidic spring mats and described as a sulfide-tolerant obligately oxygenic phototroph [26], which is consistent with activity only under conditions allowing for OP. 16S rRNA analysis also revealed that *Annamia* was the dominant genus throughout the experiment. Using microscopy, we found only four cyanobacterial morphotypes. The vastly dominating morphotype, which was indistinguishable from the photosynthetically versatile cyanobacterium described in [54], was likely an* Annamia* species. Overall, changes in light availability and sulfide concentration also triggered changes in the transcribed cyanobacterial rRNA pool suggesting that the transitions between AP and OP result from the concerted activity of several differently adapted cyanobacteria, with the dominating versatile *Annamia* sp. still likely shaping the transition to OP and thus the change of the system from a sink to source of O_2_.

Capping the sedimentary supply of sulfide in our experiment allowed us to disentangle this supply from the production within the mat right underneath the autotrophic layers and to gain insights into the well-tuned tango of interaction in the mat’s sulfur and carbon cycle over a diel cycle. Sulfide production during the illuminated periods of the day appeared to be exclusively stimulated by OP but not AP. Organic carbon excretion during OP likely represents this link to sulfide production by sulfate/sulfur reducers, because we only detected an increase of ^13^C/^12^C in the DOC pool during this time frame. We calculated that the diffusion time of small organics (e.g., acetate using a diffusion coefficient of 1 × 10^−5^ cm^2^ s^−1^ [57]) is sufficiently short (~8 min) to explain the temporal link between organic carbon release in the photosynthetically active layer and the response in the zone of sulfide production ~1 mm away.

Our method for DOC determination did unfortunately not allow quantifying and identifying the excreted compounds. Yet, DOC release during high oxygenic photosynthetic activity and stimulation of sulfate reduction rates by these photosynthates has been observed previously [58] and has been linked to photorespiration in low nutrient (e.g., nitrate) availability environments [59]. We therefore suggest that during high light conditions, high O_2_ and low CO_2_ microenvironmental conditions favor the oxygenase activity of RuBisCo yielding 2-phosphoglycolate, which can further be metabolized to glycolate [60, 61]. This freshly fixed carbon is excreted and available for the heterotrophic community including SRB [62]. Intriguingly, we did not detect ^13^C-labeled DOC during AP despite similarly high total photosynthesis rates when OP was inhibited by DCMU and sulfide was plentiful (in the second run). Since photorespiration cannot occur under anoxic and AP-dominated conditions, our results suggest that sulfide production in the mat is primarily fueled by glycolate or other compounds released during cyanobacterial photorespiration.

The upward shift of the sulfide production zone from dark to OP conditions could suggest stratification in the SRB community, with the populations closest to the cyanobacteria taking advantage of the DOC released during OP, while the deeper populations utilizing fermentation products. We could however not confirm any DOC release in the dark, likely because rates of conversion and excretion were substantially lower than during OP or because ^13^C/^12^C-DOC analysis was selective for photosynthates. As rates remained stable in the dark, while they dropped in the light under AP conditions, dark sulfide production was still likely fueled by products of fermentation, performed by the cyanobacteria in the absence of O_2_ [63, 64]. Overall, the sulfide-producing community is thus fed with DOC by cyanobacteria day and night—just not during AP.

**Fig. 7:**
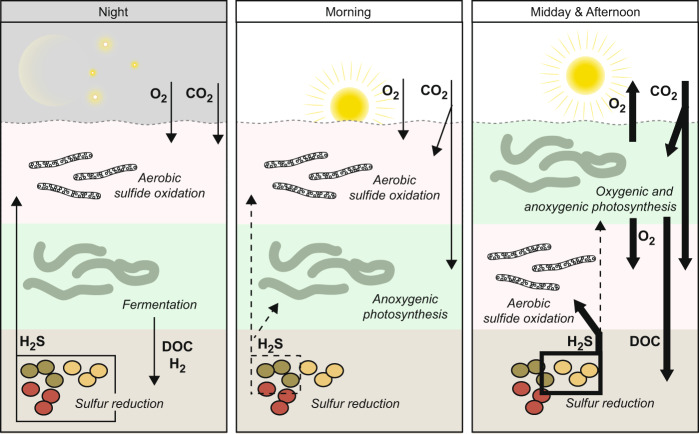
Model of carbon and sulfide flow in the Frasassi sulfidic spring mats over a 24-h cycle. Thickness of arrows indicates magnitude of fluxes between layers, with the dashed lines representing the lowest end members. Only metabolic pathways relevant to the sulfur cycle are shown.

The hypothesized interplay of metabolisms in the mat over a diel cycle is summarized in Fig. 7. Fermentation at night sustains a part of the sulfide-producing community. During OP a different population is supplied with freshly fixed and excreted organics. The mat-building cyanobacteria thus “farm” a sulfide-producing population that is adapted to the periodic availability of specific substrates. The lack of supply of small organics during AP, i.e., in the early morning, allows the cyanobacteria to switch to OP at relatively low light intensities. When DOC excretion and sulfide production rates are highest in the cyanobacterial population has already switched to OP. OP then establishes an aerobic sulfide oxidation “shield,” either by abiotic reactions or by SOB, before stimulation of sulfide production that would favor AP or even cause toxic sulfide levels for OP. This cascade overall minimizes the fraction of the day during which photosynthesis is dominated by AP. Intriguingly, the spatial separation of functional groups in combination with mass transfer resistance plays an important role in this scenario. Namely, the diffusion time of excreted DOC imposes a delay that allows for the emergence of local OP-driven O_2_ gradients that then act as the “sulfide shield.” This effect is even more pronounced because the small spatial scales allow for structural rearrangements (by SOB migration) before sulfide production is stimulated.

Overall, the Frasassi sulfidic spring mats have given unpreceded insights into the competitive advantage of photosynthetic versatility in cyanobacteria and the impact of versatility on O_2_ export. An appreciation of scale, mass transfer, and diel light dynamics are crucial to understand this advantage. If the cyanobacteria were sulfide-resistant obligate oxygenic phototrophs, for instance, DOC excretion and thus SRB stimulation would follow the light dynamics. Irradiance, however, has to be high enough to allow for OP rates that are sufficient to enable formation of the O_2_ barrier to scavenge the additional sulfide. The versatile cyanobacteria achieve this by postponing OP, while still being photosynthetically active at low light by performing AP. Similarly, excretion of DOC during AP would induce immediate response of sulfide production and thus a positive feedback loop, with reduced O_2_ export. Photosynthetic versatility, with DOC excretion limited to OP, thus enables the cyanobacteria to actively modulate the sulfur cycle. This finally facilitates out-competition of obligate anoxygenic phototrophs and net O_2_ production, despite multidirectional high sulfide supply. Specifically, sulfide is mainly supplied when the production rate of O_2_ is already high, pushing the redox cline downward and favoring SOB over anoxygenic phototrophs. In principle, the mechanism of sulfur cycle manipulation could also occur in a benthic system with overlapping photosynthetic and sulfide-producing zones. However, the delay in sulfide production after onset of OP, which is related to spatial separation of functional groups and mass transfer resistance, allows for a faster transition to OP and thus further increased O_2_ export.

Solute transfer between different functional groups in microbial mats occurs at temporal scales that allow for interactions and feedback loops within a diel cycle. These inherent properties of mats enable the differential effect of AP and OP on sulfide production. Such feedback effects are not intuitive and in stark contrast to the model introduced by Johnston et al. [3], which predicts a positive feedback loop between sulfide production and AP in the pelagic realm. This is because solute transfer between redox interfaces in the oceans’ water column occurs on substantially longer timescales and because the carbon cycle is coupled mainly by the transfer of particulates. Feedback effects consequently arise based on integrated activity over complete day–night cycles in the photic zone. Clearly, when the main mode of transport is diffusion, when scales allow “communication” across the redox zonation within a diel light cycle, and when AP and OP occur within a single functional group, different feedback mechanisms arise. Most astonishingly, O_2_ export in the sulfidic environment studied here is maximized by the cyanobacteria’s ability to perform AP during low light conditions. The capability to perform both AP and OP in a benthic environment thus allowed the cyanobacteria to modulate and even invert the positive feedback effects in the sulfur cycle predicted for the water column by Johnston et al. While the evolution of AP likely predated OP, the evolutionary history of AP within cyanobacteria remains uncertain, mainly because the sulfide oxidizing enzyme SQR is characterized by a history of intense horizontal gene transfer [7, 12, 65, 66]. As opposed to obligate anoxygenic phototrophs, cyanobacteria might have therefore acquired the capability to perform sulfide-driven AP after the evolution of OP [12] and this trait might have only been widespread in the Proterozoic when sulfide became an electron donor of global relevance. Our data highlights that the evolutionary onset of versatility in the form of SQR acquisition might have substantially reshaped the balance between aerobic and anaerobic remineralization processes and boosted O_2_ export of microbial mats. Considering the uncertainties in the control of euxinia and potential triggers of Earth’s major redox transitions, these findings emphasize the need to understand more about the evolution of cyanobacterial AP and the global extent of benthic photosynthesis over Earth’s history [7, 12, 67].

## Supplementary information

Supplementary Information

Supplementary Table S2
