# Supplementary material for: Versatile cyanobacteria control the timing and extent of sulfide production in a Proterozoic analog microbial mat
Source: ISME J. 2020 Aug 7;14(12):3024–37. doi: 10.1038/s41396-020-0734-z (PMC7784965; doi:10.1038/s41396-020-0734-z)
Supplement: Supplementary file 1 — Supplementary Information [file 41396_2020_734_MOESM1_ESM.pdf]

Supplementary Information

Versatile cyanobacteria control the timing and extent of sulfide production in a Proterozoic analogue microbial mat

Judith M. Klatt*[1], Gonzalo V. Gomez-Saez[2,3], Steffi Meyer[1,4], Petra Pop Ristova[2], Pelin Yilmaz[5], Michael S. Granitsiotis[6,7,8], Jennifer L. Macalady[9], Gaute Lavik[10], Lubos Polerecky[1,11], Solveig I. Bühring[2]

[1] Microsensor Group, Max Planck Institute for Marine Microbiology, Bremen, Germany
[2] Hydrothermal Geomicrobiology, MARUM, University of Bremen, Germany
[3] present address: Alfred Wegener Institute - Helmholtz Centre for Polar and Marine Sciences, Bremerhaven, Germany
[4] present address: Thünen Institute of Baltic Sea Fisheries, Thünen Institute, Rostock, Germany
[5] Microbial Physiology Group, Max Planck Institute for Marine Microbiology, Bremen, Germany
[6] Research Unit Environmental Genomics, Helmholtz Zentrum Munich, Germany
[7] Department of Environmental Engineering, University of Patras, Agrinio, Greece
[8] present addresses: DOE, Joint Genome Institute, Lawerence Berkeley National Lab, CA, USA
[9] Pennsylvania State University, University Park, State College, PA, USA
[10] Biogeochemistry Group, Max Planck Institute for Marine Microbiology, Bremen, Germany
[11] Department of Earth Sciences—Geochemistry, Faculty of Geosciences, Utrecht University, Utrecht, The Netherlands

* Correspondence to: jklatt@mpi-bremen.de

Supplementary Methods 2
RNA extraction and library preparation 2
16S rRNA analysis 3
FA-SIP 4
Supplementary Figures 6
Supplementary Tables 10

Supplementary Methods

RNA extraction and library preparation

Samples for 16SrRNA sequencing were immediately stabilized by collection of ~2 mL mat into RNAlater (Ambion) in 15 mL sterile conicals to a final volume of 10 mL. After a 15 min incubation time at room temperature, samples were transported and stored at -20°C. Prior extraction, samples were thawed at room temperature and centrifuged at 2,000 x *g* for 30 min at 4°C. The supernatant was discarded by aspiration. Samples were extracted with FAST RNA Pro Soil direct kit (MP Bio). Lysis buffer was directly added into the falcon tube and all the pellet was transferred to lysing matrix E tube with cut filtered tips. Samples were mixed by vortexing and then were beat for 40 s/6.0, twice with a 3 min pause on ice. Downstream steps were according to the manufacturer protocol. Precipitation of nucleic acids was overnight at -20°C, followed by centrifugation at 14,000 x *g* at 4°C for 30 min. Pellets were washed twice with 70% ice-cold ethanol. After a brief drying at room temperature to remove all residual ethanol, pellets were dissolved in 1000µl TE buffer (pH 7.5). DNA was digested with RQ1 RNase free DNase (Promega), with the addition of 40 units ribonuclease inhibitor (RNasin, Promega) according to the manufacturer instructions. After digestion, RNA was isolated with a standard RNA PCI/CI purification kit (ROTH). Precipitation of RNA was overnight at -20°C with Isopropanol/Na-Acetate, followed by centrifugation at 14,000 *x* g at 4°C for 30 min. Pellets were washed twice with 70% ice-cold ethanol. After a brief drying at room temperature to remove all residual ethanol, pellets were dissolved in TE buffer (pH 7.5). Samples went through a second DNase digestion followed by a purification with a RNeasy Minelute kit (Qiagen). RNA was eluted with 30µl TE buffer (pH 7.5), passed through the column twice. RNA quality was checked with bioanalyzer using RNA chip Nano (Agilent) and Nanodrop (Thermo). Quantification of RNA was performed with Ribogreen quantification kit (Invitrogen) according to the manufacturer

protocol. Pyrosequencing libraries were constructed as described previously [49] with modification. Briefly, the Ultrafast III qRT-PCR kit (Agilent) was used with Ba27f/519r primers, with initial 20 min at 45°C for reverse transcription. Each sample reaction was performed in triplicate. To ensure absence of residual DNA, for each sample was performed a reaction omitting reverse transcriptase. Additionally, for each sample a negative control was used. All triplicate amplicons for each sample were pooled and purified with Nucleospin kit (Macherey-Nagel) according to the manufacturer. Purified libraries were evaluated with Bioanalyzer on 7500 DNA chip and quantified with Picogreen dsDNA quantification kit (Invitrogen) Libraries were pooled in an equal molar ratio of 10^9 molecules μL^{-1}.

16S rRNA analysis

The next-generation sequencing analysis pipeline of the SILVA project (available at www.arb-silva.de/ngs) [50] allowed sequence and alignment quality-based filtering of the amplicons, to obtain aligned sequences, and a taxonomic classification to identify sequences belonging to taxa of interest. The quality cut-offs used were 50 for alignment and sequence quality, and classification cut-off of (percent query coverage + percent alignment identity)/2 > 95% was used to assign sequences to taxa. "NP" reads were not included in any further analysis due to the low sequencing output (Table S3). 12,058 cyanobacterial and 1428 deltaproteobacterial aligned sequencing reads were selected for further oligotyping analysis. Following the entropy analysis, oligotyping was performed with 26 and 25 components using the version 2.1 (available from https://meren.github.io/projects/oligotyping/), for cyanobacteria and deltaproteobacteria, respectively. To reduce noise, we imposed requirements that each oligotype must (i) appear in at least one sample, and (ii) have a most abundant unique sequence with a minimum abundance of 2. Final number of quality-controlled oligotypes for cyanobacteria and deltaproteobacteria were 75 and 11, respectively. Oligotype representatives were further checked for their taxonomic

affiliations by adding them to the SILVA RefNR 132 guide phylogenetic tree using ARB-parsimony addition tool [51]. Four oligotypes from Deltaproteobacteria had disagreements with the SILVA ngs classifications, and were eliminated from further downstream analysis (Supplementary Table S3). Further processing of the data was performed in R environment for statistical computing (https://www.R-project.org/), using package phyloseq (version: 1.19.1) [52]. In phyloseq, oligotypes were further filtered by removing those oligotypes not observed more than twice in at least 20 % of the samples.

Sequence data has been deposited in the European Nucleotide Archive (ENA) at EMBL-EBI under accession number PRJEB38493 (https://www.ebi.ac.uk/ena/data/view/PRJEB38493) using the data brokerage service of the German Federation for Biological Data (GFBio) [53]. Sequences deposited under accession numbers LR828747-LR834763 correspond to the mat sample taken at Main Spring prior to the experiment. Sequences obtained from samples taken during the first flow chamber run are deposited under LR856501-LR860505 (dark), LR849684-LR853051 (19 µmol photons m^{-2} s^{-1}), LR834764-LR841325 (first exposure to 315 µmol photons m^{-2} s^{-1}) and LR853052-LR856500 (after addition of DCMU) (Fig. 3c). LR844902-LR849683 and LR841326-LR844901 correspond to samples taken during the second incubation in the dark and during exposure to 315 µmol photons m^{-2} s^{-1} (Fig. 4c), respectively.

FA-SIP

The total lipids extracts (TLE) of freeze dried mat samples were obtained following a modified procedure [34]. Namely, combusted sand (30-40 g per sample) was used to adapt this method originally designed for lipid extraction in sediments. The extraction consists of four steps using dichloromethane/methanol with phosphate- and trichloroacetic acid buffers (each twice). 2-methyl-octadecanoic acid was used as internal standard.

After removal of elemental sulfur, an aliquot of the TLE was saponified [36]. The procedure includes a base saponification using potassium hydroxide in methanol, base extraction of the neutral lipids and acid extraction of the free fatty acids.

FA methylesters were identified by gas chromatography - mass spectrometry (GC-MS) combining an Agilent 6890N gas chromatograph with an Agilent 5973N mass selective detector. The capillary column was Restek Rtx®-5MS silica column with 30 m of length, 0.25 mm of internal diameter and 0.25 µm of film thickness. The GC operating conditions were as follows: 2 µL sample volume were injected at 60 °C for 1 min. Temperature was increased from 60 to 150 °C at 10 °C min^{-1}, then to 320 °C at 4 °C min^{-1}. The total running time was 72.5 min. Helium was used as carrier gas with a flow-rate of 1.0 ml min^{-1}. The full scan electron impact mass spectra were recorded at a range of 50-700 m/z. FA were quantified by gas chromatography coupled to a flame ionization detector (GC-FID) using same oven operating conditions as for the GC-MS except for a total running time of 60 min and the use of squalene as injection standard to check for internal precision. For the carbon isotopic compositions measured on GC-isotopic ratio-MS (GC-ir-MS; Thermo Scientific Trace GC Ultra with Thermo Scientific Delta V Plus IRMS), CO_2 was used as reference gas with a ratio of -35.25 ‰.

Supplementary Figures

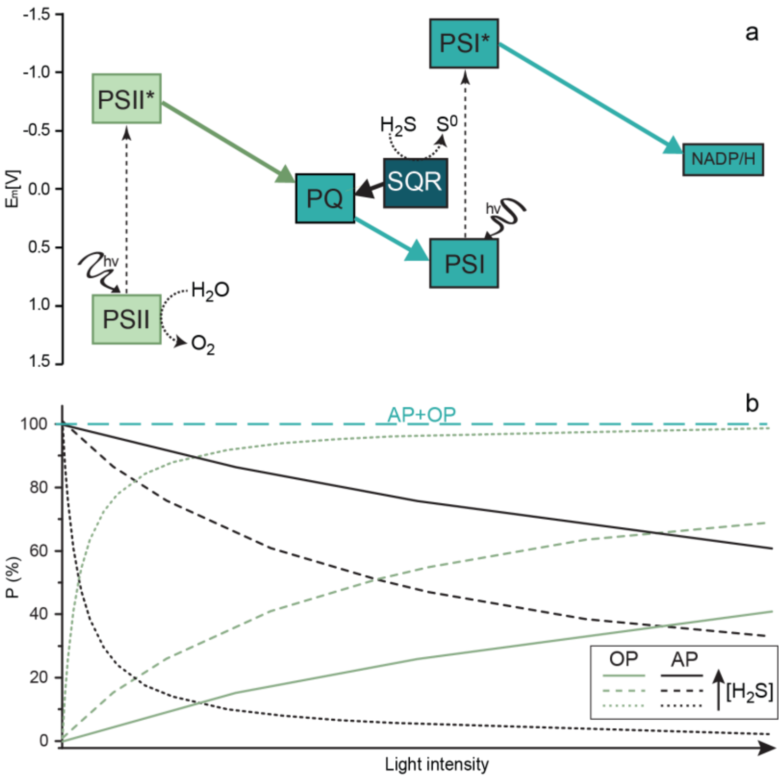

Figure S1: Photosynthetic electron transport chain in versatile cyanobacteria (a) and the resultant partitioning between anoxygenic and oxygenic photosynthesis (AP and OP) dependent on light intensity and sulfide concentration (b), modified from [12, 13, 54]. Sulfide:quinone:reductase (SQR) oxidizes H_2S and donates electrons into the plastoquinone pool (PQ). Plastoquinone is also an electron acceptor of photosystem II (PSII). Thus, anoxygenic and oxygenic electron transport intersect in the PQ pool. The sum of photosynthetic electron transport rates (AP+OP) is limited by light energy harvested in photosystem I (PSI). The partitioning between AP and OP depends on both light energy harvested in PSII and H_2S availability as conceptually illustrated in (b), and varies among cyanobacteria based on the respective apparent affinity of their SQR and PSII to PQ. The conceptual regulation of partitioning shown in (b) is derived from measurements in cyanobacterial mats, likely dominated by *Annamia* sp based on morphology, from the Frasassi sulfidic springs and modeling of electron transport rates [54].

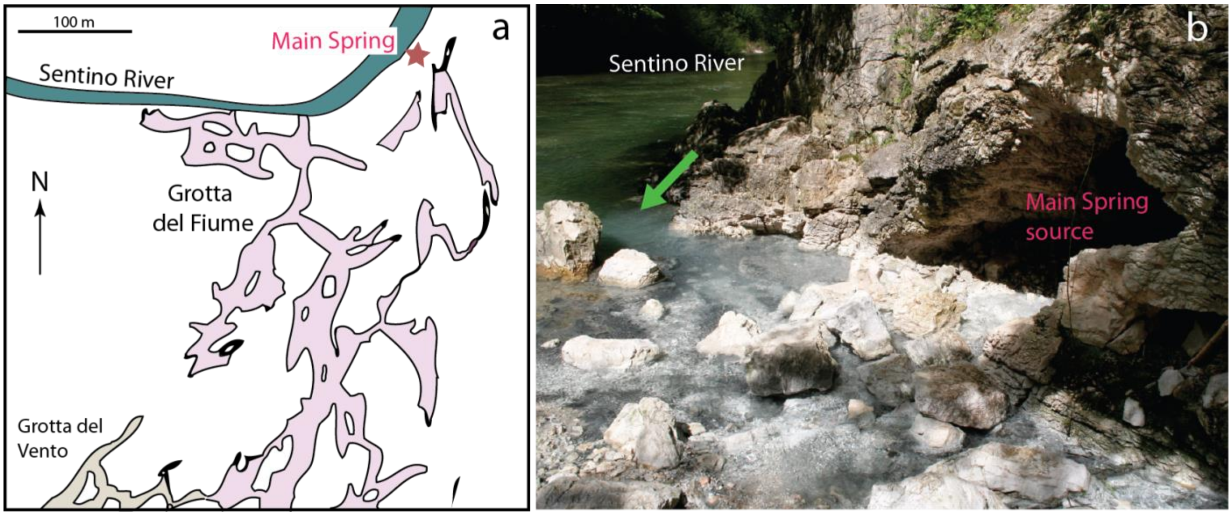

Figure S2: Map of the Northern part of the Frasassi Cave system (a, modified from [23]) and photo of Main Spring and sampling location (b). The caves form on the Eastern slope of the Apennine mountains around a canyon cut by the Sentino River, the Frasassi Gorge. The main aquifer of the Frasassi area is enriched in sulfate and sulfide, after perculation through an organic-rich Triassic anhydrite formation, and is driving cave formation by promoting chemolithotrophic sulfide oxidation, resultant sulfuric acid generation and lime stone dissolution. The sulfidic groundwater of Main Spring rises through alluvial deposits before discharging close to the Sentino river. Along the flow path, both chemosynthetic (white appearance of sediment in (b)) and photosynthetic microbial life flourishes. In (b), the sampling area of mat for the flow chamber experiments is indicated by the arrow.

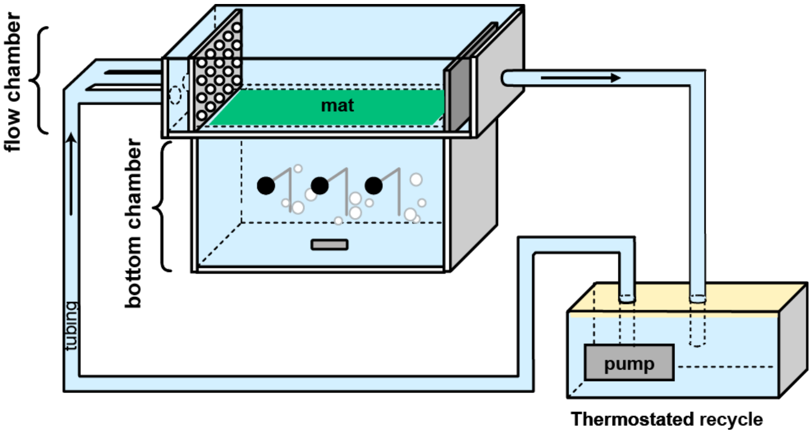

Figure S3: Schematic diagram of the experimental flow chamber.

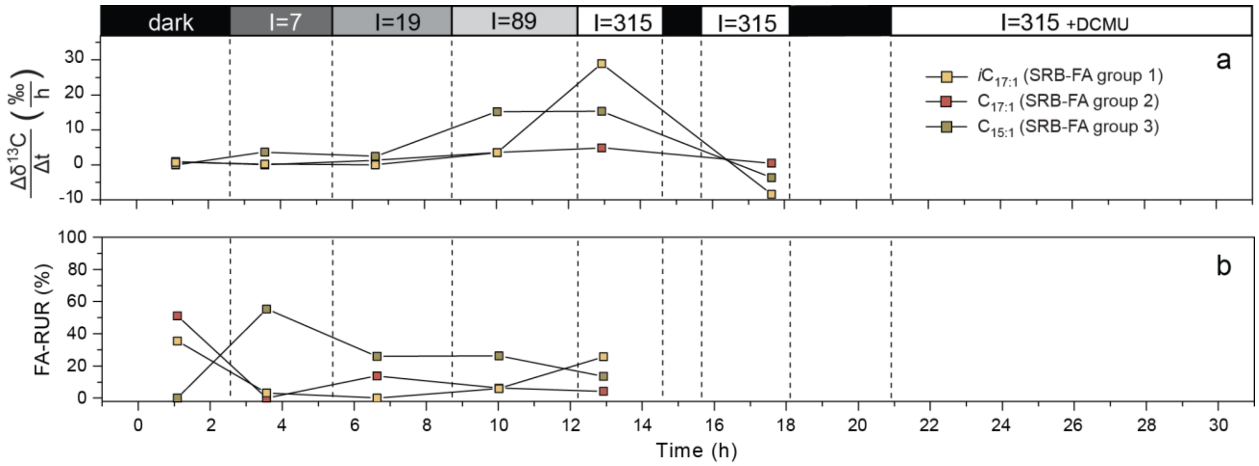

Figure S4: Examples of representative fatty acids amongst the SRB-FA sub-groups clustered based on their increase/decrease in ^{13}C uptake rate during incubation 1. Panel (a) is the uptake rate, while in (b) the relative contribution of the specific FA to the total uptake rate of the complete SRB-FA pool is shown.

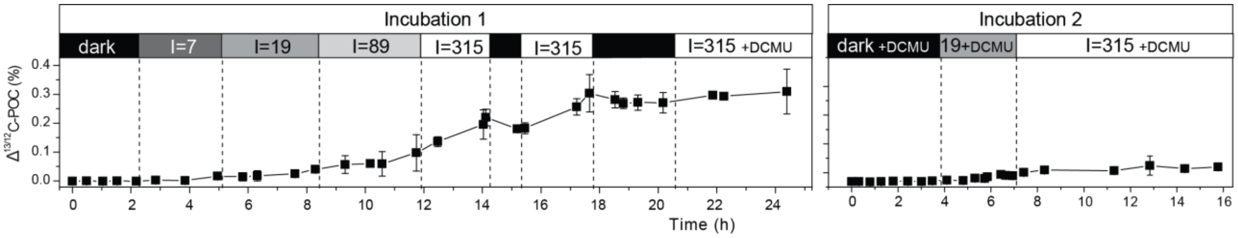

Figure S5: $^{13/12}$C of assimilated CO_2, measured in subsamples taken during the first and second incubation, relative to the first timepoint of the respective incubation.

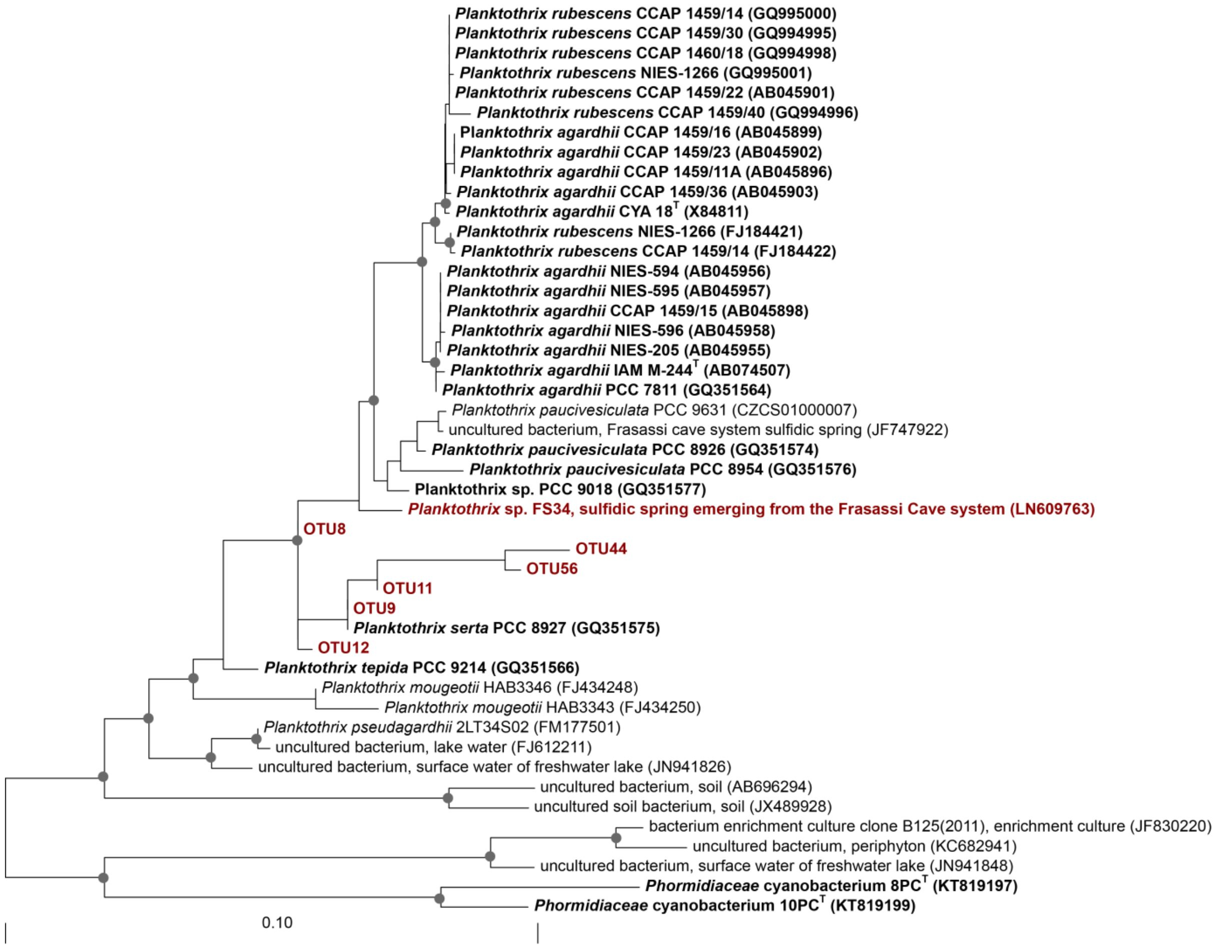

Figure S6: Taxonomic affiliation of cyanobacterial oligotype representatives from this study and *Planktothrix* sp. FS34, isolated from the Frasassi sulfidic springs [26], sequence. The SILVA RefNR 132 guide phylogenetic tree was generated using the ARB-parsimony addition tool.

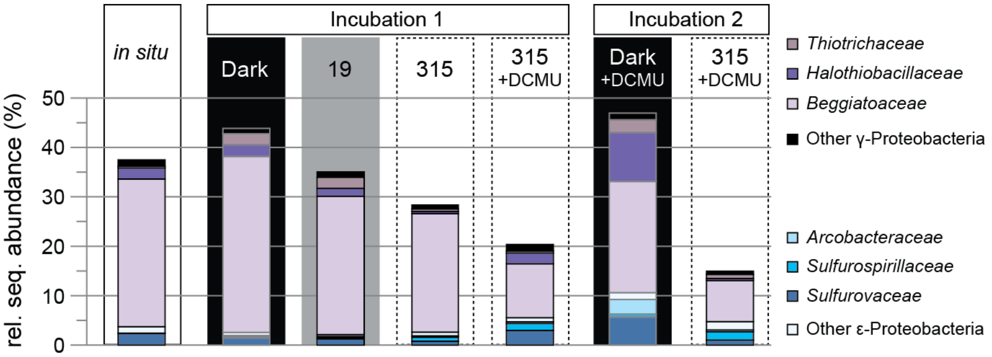

Figure S7: Relative abundance of potentially sulfur oxidizing families amongst the Gamma- and Epsilonproteobacteria in the mat *in situ* in Main Spring, and during the first and second incubation. The incubation conditions are indicated in the top panels.

Supplementary Tables

Table S1: Physico-chemical parameters measured in the water column at the sampling site in Main Spring

S_{tot} (µM)	O_2 (µM)	pH	T (°C)
68	23	7.3	14

Table S2: Fluxes of O_2 and S_{tot} measured in 1–3 replicate spots in the first and second run of the flow chamber experiment together with the theoretical stoichiometry of aerobic sulfide oxidation (aSOx).Concentrations are in µM. Fluxes including depth integrated gross rates (DIR) are in mmol m^{-2} h^{-1} and shown as negative when consumptive. Shaded rows refer to measurements in the mat spot where gross rates were assessed. These rates are plotted in Figure 1.

	Timepoint; condition	S_{tot} flux by aSOx[1]	O_2 flux by aSOx [2]	S_{tot}:O_2[3]	pH[4]	[H_2S][4]	[O_2][4]	Stoichiometry of aSOx coupled to CO_2 fixation[5]	CO_2 flux by aSOx	Net flux by AP[6]	Gross DIR of AP[6]	Net flux by OP[7]	Gross DIR of OP[7]	S_{tot} flux by SRB
1	-7h; dark	-0.158	-0.197	0.803	7.3	29	9	$H_2S + 1.245\ O_2 + 0.415\ CO_2 + 0.189\ H_2O \rightarrow 0.226\ S^0 + 0.774\ SO_4^{2-} + 0.415\ CH_2O + 1.548\ H+$	-0.066	0	0	0	0	0.586
		-0.236	-0.329	0.717	7.3	40	6	$H_2S + 1.395\ O_2 + 0.470\ CO_2 + 0.379\ H_2O \rightarrow 0.090\ S^0 + 0.910\ SO_4^{2-} + 0.470\ CH_2O + 1.819\ H+$	-0.111	0		0		0.608
	0.2 h; dark	-0.131	-0.164	0.801	7.3	24	7	$H_2S + 1.248\ O_2 + 0.415\ CO_2 + 0.191\ H_2O \rightarrow 0.224\ S^0 + 0.776\ SO_4^{2-} + 0.415\ CH_2O + 1.551\ H+$	-0.054	0	0	0	0	0.567
		-0.211	-0.293	0.721	7.3	29	6	$H_2S + 1.387\ O_2 + 0.465\ CO_2 + 0.367\ H_2O \rightarrow 0.098\ S^0 + 0.902\ SO_4^{2-} + 0.465\ CH_2O + 1.803\ H+$	-0.098	0		0		0.610
		-0.179	-0.267	0.672	7.3	33	5	$H_2S + 1.488\ O_2 + 0.502\ CO_2 + 0.495\ H_2O \rightarrow 0.007\ S^0 + 0.993\ SO_4^{2-} + 0.502\ CH_2O + 1.987\ H+$	-0.090	0		0		0.577
	2.2 h; dark	-0.137	-0.173	0.796	7.3	23	7	$H_2S + 1.256\ O_2 + 0.418\ CO_2 + 0.200\ H_2O \rightarrow 0.217\ S^0 + 0.783\ SO_4^{2-} + 0.418\ CH_2O + 1.565\ H+$	-0.057	0	0	0	0	0.577
		-0.232	-0.307	0.755	7.3	29	6	$H_2S + 1.325\ O_2 + 0.443\ CO_2 + 0.288\ H_2O \rightarrow 0.155\ S^0 + 0.845\ SO_4^{2-} + 0.443\ CH_2O + 1.690\ H+$	-0.103	0		0		0.597
		-0.182	-0.261	0.698	7.3	32	5	$H_2S + 1.433\ O_2 + 0.482\ CO_2 + 0.425\ H_2O \rightarrow 0.057\ S^0 + 0.943\ SO_4^{2-} + 0.482\ CH_2O + 1.886\ H+$	-0.088	0		0		0.590
	5.0 h; 7 µE	*-0.122*	-0.182	0.671	7.5	10	7	$H_2S + 1.491\ O_2 + 0.509\ CO_2 + 0.509\ H_2O \rightarrow 1\ SO_4^{2-} + 0.509\ CH_2O + 2\ H+$	-0.062	-0.348	-0.379	0	0	0.452
		-0.148	-0.221	0.671	7.5	12	6	$H_2S + 1.491\ O_2 + 0.509\ CO_2 + 0.509\ H_2O \rightarrow 1\ SO_4^{2-} + 0.509\ CH_2O + 2\ H+$	-0.075	-0.349		0		0.401
		-0.119	-0.178	0.670	7.5	7	5	$H_2S + 1.493\ O_2 + 0.507\ CO_2 \rightarrow 1\ SO_4^{2-} + 0.507\ CH_2O + -0.506\ H_2O + 2\ H+$	-0.060	-0.387		0		0.380
	8.3 h; 19 µE	*-0.084*	-0.125	0.670	7.4	7	21	$H_2S + 1.494\ O_2 + 0.506\ CO_2 \rightarrow 1\ SO_4^{2-} + 0.506\ CH_2O + -0.506\ H_2O + 2\ H+$	-0.042	-0.825	-1.064	0.148	0.171	0.429
		-0.068	-0.102	0.670	7.4	12	12	$H_2S + 1.493\ O_2 + 0.507\ CO_2 + 0.507\ H_2O \rightarrow 1\ SO_4^{2-} + 0.507\ CH_2O + 2\ H+$	-0.034	-0.770		0.207		0.367
	10.1 h; 89 µE	*-0.375*	-0.560	0.669	7.4	2	81	$H_2S + 1.495\ O_2 + 0.505\ CO_2 + 0.505\ H_2O \rightarrow 1\ SO_4^{2-} + 0.505\ CH_2O + 2\ H+$	-0.189	-0.724	-0.922	1.183	0.943	0.528
	11.7 h; 89 µE (after migration)	-2.801	-1.206	2.322	8.2	2	18	$H_2S + 0.431\ O_2 + 0.118\ CO_2 \rightarrow 0.967\ S^0 + 0.033\ SO_4^{2-} + 0.118\ CH_2O + 0.849\ H_2O + 0.065\ H+$	-0.331	-0.345	-0.345	1.880	1.980	2.960
		-2.242	-0.976	2.297	8.2	7	14	$H_2S + 0.435\ O_2 + 0.125\ CO_2 \rightarrow 0.959\ S^0 + 0.041\ SO_4^{2-} + 0.125\ CH_2O + 0.834\ H_2O + 0.081\ H+$	-0.280	-0.399		1.574		2.344
		-2.901	-1.270	2.284	8.2	4	17	$H_2S + 0.438\ O_2 + 0.124\ CO_2 \rightarrow 0.959\ S^0 + 0.041\ SO_4^{2-} + 0.124\ CH_2O + 0.835\ H_2O + 0.083\ H+$	-0.360	-0.329		1.978		3.101
	14.1 h; 315 µE	-3.551	-4.032	0.881	8.2	1	15	$H_2S + 1.135\ O_2 + 0.395\ CO_2 + 0.082\ H_2O \rightarrow 0.313\ S^0 + 0.687\ SO_4^{2-} + 0.395\ CH_2O + 1.374\ H+$	-1.403	-0.301	-0.388	7.279	7.037	3.745
		-3.502	-4.270	0.820	8.2	6	14	$H_2S + 1.220\ O_2 + 0.439\ CO_2 + 0.211\ H_2O \rightarrow 0.228\ S^0 + 0.772\ SO_4^{2-} + 0.439\ CH_2O + 1.544\ H+$	-1.537	-0.360		8.351		3.810
		-3.227	-3.677	0.878	8.2	2	12	$H_2S + 1.139\ O_2 + 0.400\ CO_2 + 0.092\ H_2O \rightarrow 0.307\ S^0 + 0.693\ SO_4^{2-} + 0.400\ CH_2O + 1.385\ H+$	-1.291	-0.379		7.453		3.782
	15.2 h; dark	-0.244	-0.101	2.410	7.3	39	3	$H_2S + 0.415\ O_2 + 0.117\ CO_2 \rightarrow 0.979\ S^0 + 0.021\ SO_4^{2-} + 0.117\ CH_2O + 0.861\ H_2O + 0.043\ H+$	-0.029	0	0	0	0	3.422
		-0.293	-0.128	2.298	7.3	37	3	$H_2S + 0.435\ O_2 + 0.124\ CO_2 \rightarrow 0.960\ S^0 + 0.040\ SO_4^{2-} + 0.124\ CH_2O + 0.836\ H_2O + 0.079\ H+$	-0.036	0		0		3.987

Table S2 continued

	Timepoint; condition	S_{tot} flux by aSOx[1]	O_2 flux by aSOx [2]	S_{tot}:O_2[3]	pH[4]	[H_2S] [4]	[O_2] [4]	Stoichiometry of aSOx coupled to CO_2 fixation[5]	CO_2 flux by aSOx	Net flux by AP[6]	Gross DIR of AP[6]	Net flux by OP[7]	Gross DIR of OP[7]	S_{tot} flux by SRB
1	17.7 h; 315 µE	-3.847	-4.640	0.806	8.1	2	16	$H_2S + 1.241\ O_2 + 0.437\ CO_2 + 0.222\ H_2O \rightarrow 0.215\ S^0 + 0.785\ SO_4^{2-} + 0.437\ CH_2O + 1.570\ H+$	-1.681	-0.447	-0.616	7.230	7.454	4.737
		-4.211	-5.182	0.813	8.1	3	14	$H_2S + 1.230\ O_2 + 0.435\ CO_2 + 0.211\ H_2O \rightarrow 0.224\ S^0 + 0.776\ SO_4^{2-} + 0.435\ CH_2O + 1.553\ H+$	-1.832	-0.551		8.368		4.991
		-3.988	-4.805	0.830	8.1	3	12	$H_2S + 1.205\ O_2 + 0.424\ CO_2 + 0.177\ H_2O \rightarrow 0.247\ S^0 + 0.753\ SO_4^{2-} + 0.424\ CH_2O + 1.505\ H+$	-1.691	-0.509		8.091		4.485
	20.4 h; dark + DCMU	-0.259	-0.099	2.615	7.5	32	3	$H_2S + 0.391\ O_2 + 0.109\ CO_2 \rightarrow 1\ S^0 + 0.109\ CH_2O + 0.890\ H_2O$	-0.028	0	0	0	0	3.992
		-0.524	-0.236	2.218	7.5	41	3	$H_2S + 0.451\ O_2 + 0.132\ CO_2 \rightarrow 0.945\ S^0 + 0.055\ SO_4^{2-} + 0.132\ CH_2O + 0.813\ H_2O + 0.110\ H+$	-0.069	0		0		4.826
		-0.326	-0.179	1.822	7.5	35	3	$H_2S + 0.549\ O_2 + 0.167\ CO_2 \rightarrow 0.856\ S^0 + 0.144\ SO_4^{2-} + 0.167\ CH_2O + 0.690\ H_2O + 0.287\ H+$	-0.055	0		0		4.554
	25.2 h; 315 µE + DCMU	*-0.127*	-0.191	0.664	7.3	2	5	$H_2S + 1.505\ O_2 + 0.494\ CO_2 + 0.494\ H_2O \rightarrow 1\ SO_4^{2-} + 0.494\ CH_2O + 2\ H+$	-0.063	-2.940	-3.499	0	0	2.676
		-0.199	-0.299	0.666	7.3	4	6	$H_2S + 1.503\ O_2 + 0.497\ CO_2 + 0.498\ H_2O \rightarrow 1\ SO_4^{2-} + 0.497\ CH_2O + 2\ H+$	-0.099	-3.304		0		3.079
		-0.159	-0.239	0.665	7.3	3	6	$H_2S + 1.503\ O_2 + 0.496\ CO_2 + 0.496\ H_2O \rightarrow 1\ SO_4^{2-} + 0.496\ CH_2O + 2\ H+$	-0.079	-3.335		0		3.169
	29.4 h; 315 µE + DCMU	*-0.094*	-0.142	0.662	7.2	1	7	$H_2S + 1.511\ O_2 + 0.489\ CO_2 + 0.489\ H_2O \rightarrow 1\ SO_4^{2-} + 0.489\ CH_2O + 2\ H+$	-0.046	-0.711	-0.879	0	0	0.339
		-0.114	-0.171	0.664	7.2	3	6	$H_2S + 1.507\ O_2 + 0.493\ CO_2 + 0.492\ H_2O \rightarrow 1\ SO_4^{2-} + 0.493\ CH_2O + 2\ H+$	-0.056	-0.812		0	0	0.379
		-0.102	-0.153	0.663	7.2	2	6	$H_2S + 1.508\ O_2 + 0.491\ CO_2 + 0.491\ H_2O \rightarrow 1\ SO_4^{2-} + 0.491\ CH_2O + 2\ H+$	-0.050	-0.707		0		0.365
2	0 h; dark + DCMU	-0.078	-0.110	0.710	7.9	121	4	$H_2S + 1.408\ O_2 + 0.510\ CO_2 + 0.456\ H_2O \rightarrow 0.054\ S^0 + 0.946\ SO_4^{2-} + 0.510\ CH_2O + 1.892\ H+$	-0.040	0	0	0	0	3.897
		-0.111	-0.161	0.690	7.9	146	3	$H_2S + 1.449\ O_2 + 0.526\ CO_2 + 0.510\ H_2O \rightarrow 0.016\ S^0 + 0.984\ SO_4^{2-} + 0.526\ CH_2O + 1.967\ H+$	-0.058	0		0		4.187
		-0.133	-0.172	0.771	7.9	115	4	$H_2S + 1.297\ O_2 + 0.467\ CO_2 + 0.310\ H_2O \rightarrow 0.157\ S^0 + 0.843\ SO_4^{2-} + 0.467\ CH_2O + 1.685\ H+$	-0.062	0		0		3.798
	3.4 h; dark + DCMU	-0.128	-0.180	0.711	7.9	119	4	$H_2S + 1.406\ O_2 + 0.510\ CO_2 + 0.454\ H_2O \rightarrow 0.056\ S^0 + 0.944\ SO_4^{2-} + 0.510\ CH_2O + 1.888\ H+$	-0.065	0	0	0	0	3.824
		-0.361	-0.491	0.735	7.9	147	3	$H_2S + 1.361\ O_2 + 0.492\ CO_2 + 0.394\ H_2O \rightarrow 0.098\ S^0 + 0.902\ SO_4^{2-} + 0.492\ CH_2O + 1.803\ H+$	-0.178	0		0		4.173
		-0.211	-0.298	0.706	7.9	119	5	$H_2S + 1.416\ O_2 + 0.514\ CO_2 + 0.468\ H_2O \rightarrow 0.046\ S^0 + 0.954\ SO_4^{2-} + 0.514\ CH_2O + 1.907\ H+$	-0.108	0		0		3.997
	6.8 h; 19 µE + DCMU	*-0.159*	-0.233	0.682	8.0	59	4	$H_2S + 1.465\ O_2 + 0.534\ CO_2 + 0.534\ H_2O \rightarrow 1\ SO_4^{2-} + 0.534\ CH_2O + 2\ H+$	-0.085	-3.535	-4.134	0	0	4.090
		-0.144	-0.211	0.682	8.0	48	3	$H_2S + 1.467\ O_2 + 0.533\ CO_2 + 0.533\ H_2O \rightarrow 1\ SO_4^{2-} + 0.533\ CH_2O + 2\ H+$	-0.077	-3.824		0		3.988
		-0.189	-0.277	0.683	8.0	63	5	$H_2S + 1.465\ O_2 + 0.535\ CO_2 + 0.535\ H_2O \rightarrow 1\ SO_4^{2-} + 0.535\ CH_2O + 2\ H+$	-0.101	-3.691		0		4.013
	10.2 h; 315 µE + DCMU	*-0.255*	-0.375	0.680	8.0	12	4	$H_2S + 1.472\ O_2 + 0.528\ CO_2 + 0.528\ H_2O \rightarrow 1\ SO_4^{2-} + 0.528\ CH_2O + 2\ H+$	-0.135	-2.522	-3.018	0	0	2.599
		-0.162	-0.238	0.680	8.0	15	3	$H_2S + 1.471\ O_2 + 0.528\ CO_2 + 0.528\ H_2O \rightarrow 1\ SO_4^{2-} + 0.528\ CH_2O + 2\ H+$	-0.085	-2.733		0		2.528
		-0.128	-0.188	0.680	8.0	11	5	$H_2S + 1.471\ O_2 + 0.528\ CO_2 + 0.528\ H_2O \rightarrow 1\ SO_4^{2-} + 0.528\ CH_2O + 2\ H+$	-0.067	-2.832		0		2.987
	15.9 h; 315 µE + DCMU	*-0.209*	-0.309	0.677	7.9	8	5	$H_2S + 1.477\ O_2 + 0.523\ CO_2 + 0.523\ H_2O \rightarrow 1\ SO_4^{2-} + 0.523\ CH_2O + 2\ H+$	-0.109	-0.911	-1.256	0	0	0.386
		-0.153	-0.226	0.677	7.9	7	4	$H_2S + 1.478\ O_2 + 0.522\ CO_2 + 0.522\ H_2O \rightarrow 1\ SO_4^{2-} + 0.522\ CH_2O + 2\ H+$	-0.080	-0.990		0		0.361
		-0.144	-0.213	0.678	7.9	9	6	$H_2S + 1.476\ O_2 + 0.524\ CO_2 + 0.524\ H_2O \rightarrow 1\ SO_4^{2-} + 0.524\ CH_2O + 2\ H+$	-0.075	-0.805		0		0.320

[1]In the dark Stot fluxes for aerobic sulfide oxidation (aSOx) were calculated as the difference between the upward flux from the zone underneath into the SOB layer and the upward flux from the SOB layer towards the water column. For light conditions, before downwards migration of the SOB, S_{tot} fluxes (italic) attributed to SOB were predicted based on the O_2 flux into the SOB layer and the lowest possible S_{tot}:O_2 consumption ratio assuming an efficiency of 16.9%. After migration of SOB under the cyanobacterial layer, upward fluxes of S_{tot} were fully attributed to the SOB activity.

[2]In the dark and presence of DCMU, the O_2 flux from the water column into the mat was fully assigned to O_2 consumption by aSOx. In the light, before migration, O_2 consumption was calculated as the difference between the upward flux of O_2 from the cyanobacterial layer into the SOB layer and the flux from/into the water column. After migration, the downward flux from the cyanobacterial layer into the SOB layer underneath was attributed to aSOx.

[3]Italic ratios are predicted based on the O_2 flux, an efficiency of autotrophic sulfide oxidation of 16.9%, and complete sulfide oxidation to SO_4^{2-}.

[4]Concentrations of O_2 (in µM) and H_2S (in µM), and pH were calculated by averaging the values measured in the SOB layer and used as input parameters to calculate the stoichiometry of aerobic sulfide oxidation.

[5]The stoichiometry of aerobic sulfide oxidation coupled to CO_2 fixation was calculated either based on an efficiency of 16.9%, local reactant concentrations (see footnote 4), and the S_{tot}:O_2 ratio, or, if the S_{tot}:O_2 was unknown, based on an efficiency of 16.9%, local reactant concentrations, and the assumption that the only product of sulfide oxidation was SO_4^{2-}.

[6] in mmol S_{tot} m^{-2} h^{-1}

[7] in mmol O_2 m^{-2} h^{-1}

Table S3: *Available as a separate supplementary file.* Results of 16SrRNA sequence analysis using the next-generation sequencing analysis pipeline of the SILVA project (available at www.arb-silva.de/ngs), including sequence classifications and oligotyping results (https://github.com/merenlab/oligotyping). MID08 was obtained from a mat sample taken at Main Spring prior to the experiment. MID17, MID15, MID11 and MID16 were obtained from samples taken during the first flow chamber run in the dark, during exposure to 19 µmol photons m^{-2} s^{-1}, during first exposure to 315 µmol photons m^{-2} s^{-1} and after addition of DCMU (Fig. 3c). MID14 and MID13 correspond to samples taken in the dark and during exposure to 315 µmol photons m^{-2} s^{-1} in the second experiment (Fig. 4c).
